# Sustainable Digital Economy Through Good Governance: Mediating Roles of Social Reforms and Economic Policies

**DOI:** 10.3389/fpsyg.2021.773022

**Published:** 2021-11-24

**Authors:** Tang Xianbin, Wu Qiong

**Affiliations:** Xinjiang University, Ürümqi, China

**Keywords:** good governance, economic policies, social reforms, sustainable digital economy, e-commerce

## Abstract

The most powerful and crucial concept today is a sustainable digital economy. This research is aimed to investigate the predictors of a sustainable digital economy in China. In addition, the mediating roles of social reforms and economic policies were investigated between good governance and a sustainable digital economy. This cross-sectional research considered partial least square–structural equational modeling (PLS-SEM) as an analysis technique. The data were collected from 317 managerial staff of the e-commerce industry in China via a self-structured questionnaire. A random sampling technique was applied in the data collection process. Results showed that good governance positively impacts the sustainable digital economy, social reforms, and economic policies. Additionally, an increase in social reforms and economic policies led to a sustainable digital economy in China. Social reforms and economic policies partially mediated the relationship between good governance and a sustainable digital economy. This research contributes to the body of knowledge by identifying components of a sustainable digital economy and examining whether good governance may aid in attaining a sustainable digital economy. Nowadays, research on the sustainable digital economy has got attention from policymakers and researchers around the globe. These outcomes suggest several ways to improve the sustainable digital economy in China. This research is not without limitations, such as cross-sectional and based on responses of the respondents. Several research avenues were discussed and can be influenced by many factors for future perspectives.

## Introduction

The notion of a sustainable digital economy is the most powerful and significant concept today, as it may lead a country out of crisis and on to a path of sustainable development and establish plans and objectives that span large-scale digital economies (Yang and Zhao, 2018). Today, digital economies encompass the rapid growth of information and communication technologies (ICTs) with the expectation that they will accomplish substantial technical advances to attain digital sustainability. In addition, globalization and the digital economy have resulted in extraordinary expansion across all private and public sectors and the creation of a worldwide accessible market. One must emphasize the importance of governments and private sectors collaborating to develop a new digital ecosystem because a connected nation may transform a digital economy with more effective and convenient private and public sectors (Ubaydullaev, 2021). Furthermore, both individually and collectively, the digital economy is reshaping conventional transactions and enabling new ones (Stojanović et al., 2016).

There is a global consensus that governance approaches are required to appropriately balance potential advantages and risks of digitalization and maintain a sustainable digital economy (Yuan et al., 2021). Different perspectives and ideas have been presented on the appropriate governance techniques required to develop digitalized economies, govern the processes, and digitalization effects (Smirnova and Rudenko, 2017; Simangunsong et al., 2019; Ubaydullaev, 2021; Yuan et al., 2021). Sustainable development is still one of the most important concerns confronting the modern world. The rapid advancement of digital systems and their growing scale and complexity, new challenges for government and industry in promoting long-term digital growth, particularly in terms of recent work, responsible consumption, reduced inequalities, etc. outlined in the United Nations Sustainable Development Goals (Mickoleit et al., 2009; Thurik et al., 2019). It also seems like a major trend in environmental policy and economic growth (Linkov et al., 2018).

Moreover, good governance describes the process of public administration that optimizes public interests. One of its key characteristics is a type of collaborative administration of public life conducted by both citizens and the state and a new connection between civil society and the political State (MacDonald and Hasmath, 2020). To summarize all viewpoints on good governance, we can consider its six fundamentals: transparency, rule of love, legitimacy, accountability, effectiveness, and responsiveness (Mickoleit et al., 2009). Good governance can only be accomplished in a free and democratic political system since it is impossible to attain without them. Previous research studies have shown that individuals are happier with their lives in nations with higher levels of governance quality. Because good governance is an effective and constructive collaboration between citizens and the State, the foundation to its sustainability lies in authorities engaging in political administration (Linkov et al., 2018). Good governance is also an important component of effective economic policy since it contributes to the maintenance of an environment that promotes robust and equitable growth (MacDonald and Hasmath, 2020).

A reform movement is a social movement that seeks to modify or enhance specific elements of society progressively, and it does not advocate for drastic or fundamental improvements. While contrast, revolutionary movements attempt to transform society as a whole (Kondratiuk-Nierodzińska, 2016; Keping, 2018). Currently, China is one of the world’s largest economies, with the largest economic scale and worldwide influence. The Chinese government is emphasizing the development model and efficiency of internal governance. Because for major countries, particularly for China, effective internal governance is the cornerstone of any foreign strategy, and the ultimate determinant of the former is the ability of the country to reform (Mansell, 2010; Nannestad et al., 2014; Ivanova et al., 2019). This study contributes to the literature by identifying elements for a sustainable digital economy and determining whether good governance can play a key role in achieving a sustainable digital economy. This study evaluated the mediating function of China’s social reforms in understanding this relationship since reforms allow nations to achieve historic and great accomplishments and quick development and continual improvements of international status. Moreover, this paper has studied the mediating role of economic policies between good governance and sustainable economic policy for deeper understanding. Previous researchers have documented the role of economic policies in a different context, but in this context have never been studied before. Therefore, in this study, researchers understand the relationship between good governance and sustainable digital economy through the mediating role of social reforms and economic policy.

The remaining sections of this study are as follows: The second section discusses “Literature Review” on considered variables and the development of a hypothesis. The next third section is related to research “Methodology,” which is employed to test the hypothesis. Fourth section is concerned with the “Interpretation” of our empirical study. The last fifth section, “Conclusion,” concludes our results by offering future recommendations and implications.

## Literature Review

### Good Governance

Governance is the collection of all methods through which individuals and institutions, both public and private, manage their shared concerns (Avotra et al., 2021a). It is a continual process of balancing competing or divergent interests and taking coordinated action. It encompasses both official organizations and regimes with authority to compel compliance and informal agreements that individuals and institutions have agreed to or believe are in their best interests (Ott, 2011). It has four characteristics: governance is a process rather than a collection of rules or activity; the process of governance which is based on cooperation rather than control; it incorporates both the private and public sectors; and is not a formal institution but ongoing interaction (Okot-Uma and London, 2000). Essentially, governance is exerting authority to preserve order and addresses the demands of the general population within a set of parameters (Roblek et al., 2020; Dahwan and Raju, 2021). The goal of governance is to maximize the public interest through guiding, steering, and regulating actions of people via the use of various institutions and relationships. Good governance is defined as a commitment to democratic ideals, norms and practices, trustworthiness services, just and honest business, and the procedures and institutions that drive political and socioeconomic connections (Piątkowski and Binczyk, 2002; Nandal et al., 2021). According to United Nations, good governance has four principles: transparency, participation, consensus-oriented, the rule of law, effectiveness and efficiency, equity and inclusiveness, responsiveness, and accountability (Miller and Wilsdon, 2001; Fukuyama, 2013). Governance has several meanings and is used in several contexts. However, there is broad agreement that governance is related to the evaluation of governing methods in which the borders between private and public sectors become blurred (Helliwell and Huang, 2008). Government tasks are increasingly regarded as more general, generic, social concerns that political institutions and other players may handle. The notion of governance envisions a move away from well-established concepts of top-down government approach to resolving societal challenges. Thus, good governance may enhance evaluations of life directly because individuals are happier living in a setting of excellent government or indirectly since good governance allows people to attain greater levels of something relevant to their wellbeing.

### Social Reforms

A reform movement is a social movement that seeks to progressively modify or enhance specific elements of society (Kolosov et al., 2017; Glass and Newig, 2019; Estache and Foucart, 2021). A reform movement does not advocate for dramatic or fundamental reforms (Helliwell, 2014). Revolutionary movements, on the other hand, attempt to alter the whole society. Good governance influences the quality of citizen-government interactions and the quality of citizen-to-citizen interactions (Helliwell et al., 2014). One such method is by increasing social trust in general. Evidence from the literature (Cohen, 2004; Heeks and Alemayehu, 2009; Balcerzak and Bernard, 2017; Kyriacou et al., 2019) indicates that individuals live better lives in places where they believe and trust others, such as police, neighbors, coworkers, and strangers. The quality of governments entities, in turn, can enhance those views of trustworthiness (Howell et al., 2018). Countries can accomplish historic and great triumphs via social reforms, quick development, and continual improvements in global prestige (Frey and Stutzer, 2005).

### Economic Policies

Governance is one of the main reasons for the disparities in performance between countries. The digital economy offers a plethora of options for economies to achieve more equitable growth. To make the most of digital technology, a free flow of data must be encouraged, backed up by a set of rules that meet other public policy goals. There are two types of digital technologies: information technology (IT) and communication technology (CT). Artificial intelligence (AI), robots, and machine learning are examples of information technology that speed up data processing, minimize the number of tasks, and provide concentration pressure for economic activity (An et al., 2021). The use of CT will also have significant consequences for the inclusivity mandated by the sustainable development goals. While platform providers demand highly skilled personnel, and users are not required to have such skills. CT makes information, communication, and economic possibilities more accessible (Frey and Stutzer, 2005; Helliwell et al., 2018). The policy system for data governance, on the other hand, is still in its infancy; it is undeveloped and scattered among nations. one underlying issue is that the logic of economic justification for actions is not clearly defined. Policies governing data flows and data-related companies are regulated by numerous ministries and organizations, with little to no coordination (Androniceanu et al., 2020).

### Sustainable Digital Economy

Every country in the world is looking for ways to revitalize its economy, yet they all face huge challenges. Apart from many other sectors, the ICT industries have shown to be relatively robust throughout recent tumultuous times, as more individuals, businesses, and government have shifted their emphasis to the digital economy because it provides several benefits, such as low costs, speed of transactions, and international coverage (Lee et al., 2016; Roblek et al., 2020). High-and medium-growth enterprises have exceeded other sectors of the economy in terms of new business formation, share appreciation, and survival rates. The digital economy that includes digital skills and capital currently accounts for roughly 22.5% of the global economy. It still has a lot of room to grow and intertwine with the traditional economy (Cohen, 2004; Balcerzak and Bernard, 2017; Fogel and Etcheverry, 2019). The digital economy is characterized by integrating technology and the capacity to bridge the gap across digital, physical, and biological systems. It is commonly referred to as a digital information-based economy (Avotra et al., 2021a,b).

To be more precise, a digital economy encourages commodity circulation and the growth of the service industry through the interchange of digital information and online transactions. In the digitalization era, ICT tools provide a worldwide platform for individuals and organizations worldwide, allowing intercommunication and collaboration between various players (Ivanova et al., 2019). As a result, digital economy sustainability may be defined as actions that employ digital technologies creatively to meet sustainable development goals. Sustainable businesses have both self-interest and collection aims in mind, focusing on economic, environmental, and social objectives. Consequently, the influence of sustainability goals with digital technology has become essential in both corporate and governmental sectors (Paster, 2013; Naughton, 2014; Rotberg, 2014; Howell et al., 2018).

### Good Governance Theory

Good governance theory is referred to as allocation and management of resources to address collective challenges, and it occurs when a state efficiently delivers excellent public goods to its inhabitants (Stojanović et al., 2016). This necessitates evaluating states in terms of the quality and quantity of public goods they give to citizens. Three key elements of good governance are efficiency, openness, and accountability. The capacity of government to provide predictability in policy and institutional environments is known as efficiency (Smirnova and Rudenko, 2017; Ubaydullaev, 2021). Efficiency aids in the prioritization of government services to align them with the requirements of citizens. Accountability entails making each individual responsible for their actions. It refers to responsibilities and duties associated with a certain institution in public administration. Good governance fosters gender quality, protects the environment, allows residents to express personal freedom, offers instruments to alleviate poverty, fear, deprivation, and creates a safe atmosphere free of violence (Rothstein et al., 2012; Vishnivetskaya and Ablyazov, 2020; Xu et al., 2021). These principles enhance democratic institutions by ensuring frequent, free and fair elections, and a representative legislature, independent judiciary, and media (Rothstein and Eek, 2009; Urbaniec, 2015; Watanabe et al., 2018).

### Good Governance and Social Reforms

Good governance influences not just the quality of citizen-government interactions but also the quality of citizen-to-citizen interactions. Enhancing social trust, in general, is one such method (Fernando et al., 2019). According to the previous research, people enjoy better lives where they believe and can trust others, such as police, coworkers, neighbors, and strangers. Individuals are happier living in a setting of excellent government; thus, good governance may enhance life evolution directly or indirectly since good governance allows people to reach greater levels of something else (Helliwell et al., 2014). The quality of government entities, in turn, can impact those views of trustworthiness. Furthermore, natural migration experiments from countries with the lowest to highest quality institutions show that improvement in institutional quality boosts social trust and that institutional differences outweigh cultural differences in analyzing social trust levels (Glass and Newig, 2019). Based on this discussion, the study proposes its hypothesis as follows:

***H1:*** *Good governance leads to social reforms.*

### Good Governance and Economic Policies

Good governance is a major concern in public administration management. This is evident, among other things, in high demand of individuals on state organizers, both in government, legislation, and court, to organize effective governance (Helliwell, 2014). Good governance is critical to establishing and maintaining an environment that promotes roust and equitable growth and is a necessary component of effective economic policy (Cohen, 2004). Good governance is defined as a commitment to democratic ideals, norms, practices, trustworthy services, and honest business as procedures and institutions that drive political and socio-economic connections (Balcerzak and Bernard, 2017). To avoid envisioning the internet as an abstract change agent, the study agenda should incorporate political economy, particularly state-business relations, as a crucial level of analysis, taking into account historical place of a country in global digital capitalism. Currently, governments are facing with the challenge of developing a highly competitive knowledge-based economy that would reduce the development gap with technologically advanced economies (Geels and Smit, 2000; Balcerzak and Pietrzak, 2016; Fogel and Etcheverry, 2019). The experience of certain countries that have achieved the status of developed economies in recent decades confirms that a technological leap forward is not feasible without policies and institutional reforms that establish a successful digital economy (Frey and Stutzer, 2005; Helliwell et al., 2018). Based on the above discussion, the study proposed the hypothesis as follows:

***H2:*** *Good governance leads to favorable economic policies.*

### Good Governance and a Sustainable Digital Economy

Digitalization has become prevalent in every economic sector and significant aspect of society, altering our daily lives, business models, and how we act and think in policy and practice. Hence Sustainable development is the consequence of quality and quantity transformation in the economic, social, and environmental spheres under the assumptions of efficient and effective space management (Heeks and Alemayehu, 2009; Vlasov et al., 2019; Yuan et al., 2021). Digital advances had both good and bad consequences on three pillars of sustainable development society, economy, and environment at each stage of evolution. The digital economy expanded more slowly during the crisis, but its future expansion is regarded as one of the elements that can assist nations in dealing with a crisis (Cohen, 2004; Helliwell, 2014). As a result, good governance is the most important requirement for fulfilling ambitions of an individual in accomplishing goals and values of the nation and state. In this scenario, the creation and execution of a clear, suitable, and sound system of accountability are required so that the government may be implemented efficiently, responsibly, collusion, successfully, free of corruption, and nepotism (Geels and Smit, 2000; Urbaniec, 2015; Balcerzak and Pietrzak, 2016; Watanabe et al., 2018). Based on this discussion, the study proposes its hypothesis as follows:

***H3:*** *Good governance leads to a sustainable digital economy*.

### Social Reforms and a Sustainable Digital Economy

The worldwide digital transformation has affected many different elements of the economy, society, and private lives of individuals. The core concept of a digital economy is that contemporary technology supports transmissions and processing of products, lifelong learning, services, and innovation in the framework of market globalization and sustainable development (Helliwell et al., 2014; Glass and Newig, 2019). Aside from economic and social implications, the environmental effect of the digital economy requires special consideration since it is an essential component of long-term growth. Ott (2011) argues that the digital economy is present in every significant sector of society. The political agenda should be reconstructed to include concerns about the digital economy’s environmental effect. Rothstein and Eek (2009) illustrate that simplification of environmental impact studies results in unsuccessful technological futures. According to Geels and Smit (2000); Balcerzak and Bernard (2017), and Glass and Newig (2019), the digital economy alters the human-environment relationship through altering business paradigms. They promote the notion of the sustainable digital economy as a solution to environmental concerns. They examine the prospect of harnessing the creativity and energy of the digital economy for the benefit of the economy, society, and the environment. In general, digital technology has had a significant impact on value chain of nearly every industry. Based on this discussion, the study proposes its hypothesis as follows:

***H4:*** *Social reforms lead to a sustainable digital economy.*

### Economic Policies and Sustainable Digital Economy

The digital economy of apps and services has emerged as one of the world’s most significant drivers today because the internet serves as a foundation for such a digital economy (Howell et al., 2018). From AI to cloud computing, the internet of things, new Web-enabled ICT applications are set to penetrate and change the economy and social life. In digitalization, ICT facilities provide a worldwide platform for individuals and organizations all over the world, allowing intercommunication and collaboration among various actors (Heeks and Alemayehu, 2009). As a result, digital economy sustainability may be defined as actions that attempt to achieve sustainability objectives through the creative application of digital technologies. Because China is growing dominantly, the Chinese government is consciously incorporating network connection and networked technologies into the main national economic restructuring agenda of the country (Piątkowski and Binczyk, 2002; Mansell, 2010; Kolosov et al., 2017).

Economic restructuring is defined as a deliberate shift from consumption based to an innovation-driven economy because a key state goal for China at a level that had never been seen after 2008. Economies develop such economic strategies to nurture more sophisticated labor divisions, build domestic consumption capacity, and stimulate innovation and company growth (Keping, 2018; Song et al., 2021). Based on the above discussion, the study proposed the hypothesis as follows:

***H5:*** *Favorable economic policies lead to a sustainable digital economy.*

#### The Mediating Role of Social Reforms

Good governance influences the quality of citizen-government interactions and the quality of citizen-to-citizen interactions (Chen et al., 2020). Enhancing social trust, in general, is one such method. According to researchers, people enjoy better lives where they believe and can trust others, such as police, coworkers, neighbors, and strangers (Vlasov et al., 2019). The quality of governmental institutions can impact these trustworthiness judgments in turn. The contemporary world would be unimaginable without the widespread use of information technology, which has vastly improved the commercial operations of businesses while also improving the management system (Helliwell and Huang, 2008). As a result, a study in the subject of the digital economy is highly relevant since it examines a new path of economic theory and practices. The digital economy seems to be an activity in which the significant components in production are data presented in digital form, their processing and use in large volumes improve efficiency, quality, and productivity in various types of technology, storage, production, delivery, equipment, sale, and consumption of goods and services (Rothstein and Eek, 2009; Urbaniec, 2015; Watanabe et al., 2018). Since the end of the 20th century, the diffusion of digital technologies in the economy and society has resulted in a situation in which experts have begun to discuss the digital revolution, leading to scale and radical transitions of many aspects of business, providing tremendous opportunity, and penetrating all fields of the global economy (Stojanović et al., 2016; Howell et al., 2018; Ubaydullaev, 2021). Furthermore, digital platforms are frequently utilized in international practices to monitor and evaluate efficacy and efficiency of state agencies, particularly, in terms of monitoring and analyzing the quality of public services (Gnan and Masciandaro, 2020; Nandal et al., 2021; Yuan et al., 2021). The above discussion reveals that social reforms significantly mediate the relationship between good governance and a sustainable digital economy.

***H6:*** *Social reforms mediate the relationship of good governance and a sustainable digital economy.*

#### The Mediating Role of Economic Policies

The governance framework is critical for transforming growth and welfare into long-term processes. As a result, the governance structure is critical for the growth, long-term development, and equitable income distribution. Governance is critical to societal wellbeing (Srivastava, 2009; Gnan and Masciandaro, 2020). Better-governed countries are wealthier, happier, and have fewer social and environmental issues. One of the major explanations for performance variations of countries is governance (Yang and Liu, 2016; Dhir et al., 2021). The efficiency of the public sector determines the effectiveness of various policy instruments. The digital economy offers many options for economies to achieve more equitable growth (Rothstein et al., 2012). To use digital technologies, a free flow of data must be encouraged, backed up by a set of rules that meet other public policy goals. Policies governing data flow and data-related companies, on the other hand, are still undeveloped and scattered among nations (Howell et al., 2018).

The larger multidisciplinary subject of information society and ICT policy highlights the perspective of developed nations conceiving ICTs as drivers of productivity, efficiency, and promoting the western paradigm of market-led technology spread. The digital economy has grown into a multibillion-dollar industry. The connections between digitalization and industrialization are mediated by economic policy, political economics, and social dynamics (Kolosov et al., 2017). To avoid abstractly viewing the internet as a change agent, the study agenda should incorporate a critical degree of examination of political economy, particularly state-business relations, while evaluating historical place of a country in global digital capitalism (Vlasov et al., 2019). Currently, governments face the challenge of developing highly competitive expertise economies that will narrow the infrastructure gaps with economies at the technology frontier (Kyriacou et al., 2019). The experience of certain countries that have achieved the status of advanced economies in recent decades confirms that a leap in technology forward is not feasible without policies and institutional changes that result in the establishment of a successful digital economy. This shows that the economic policies significantly mediate the relationship of good governance and a sustainable digital economy because countries mostly reform their economic policies to stabilize their economic growth. Based on this discussion, this study proposed the hypothesis as follows:

***H7:*** *Favorable economic policies mediate the relationship between good governance and a sustainable digital economy.*

**FIGURE 1:**
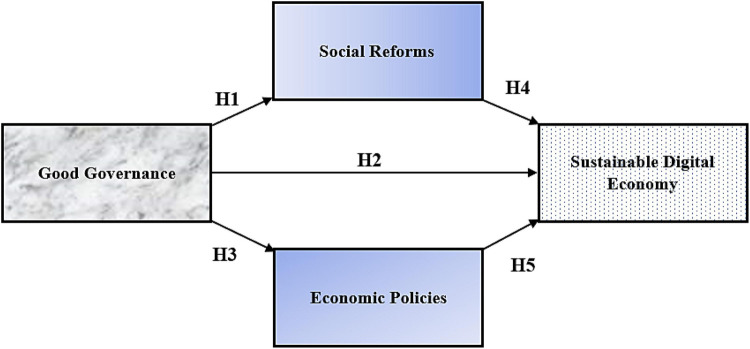
Conceptual model.

Based upon the literature review, this research was designed, and the following conceptual framework (Figure 1) was developed. The research revolves around this.

## Methodology

This study investigates the predictors of a sustainable digital economy through good governance and mediating roles of social reforms and economic policies in China. This study is cross-sectional, and a structured questionnaire was used to evaluate quantitative data. A total of 21 items were employed to develop a questionnaire for variable analysis.

The study used primary data sources, and data were collected via convenience random sampling. Researchers can obtain data from individuals who are easily available and willing to engage using convenience random sampling. Data for the survey were gathered from executives in e-commerce business of China. The total number of items will determine the sample size; hence, 317 responses will be used for analysis. A few demographic questions will be added to understand better responses, such as age, gender, education, experience, and job role. In this study, the data analysis approach was utilized partial least square–sequential equation modeling (PLS-SEM) in Smart-PLS 3.3.3. As a result, previous research measurements were used in this study to assess all the constructs of the current model.

### Instrument Development

In this research, we developed a measuring scale for all these constructs using previous indications. The responses were rated using a five-point Likert scale ranging from 1 (strongly disagree) to 5 (strongly agree). We have investigated the reliability and validity of all constructs by using confirmatory factor analysis (CFA) and exploratory factor analysis (EFA) analysis (PLS-algorithm) in Smart-PLS. We measured good governance through indicators used in a prior study (Afonso and Fernandes, 2006; Srivastava, 2009) and consist of five items. All the measurement items of social reforms were adapted from and seven items were chosen to measure social reforms (Okot-Uma and London, 2000; Miller and Wilsdon, 2001; Linkov et al., 2018). While this research made five items scale for economic policy based on previous research (Tsoukas and Papoulias, 1996; Simangunsong et al., 2019). Likewise, a sustainable digital economy is measured through indicators used in the previous studies (Kolosov et al., 2017; MacDonald and Hasmath, 2020) with four items. In total, 21 items were sued to measure four constructs in the current research model.

## Data Analysis

Table 1 shows a summary of respondents to whom data were gathered. After screening the data, a total of 317 samples have been used for the data analysis purposes; among them, 160 (50.5%) were male and 157 (49.5%) were female. Most of them held master’s degree qualifications (56.2%) and the rest of them held bachelor’s or below (15.1%), doctorate’s (17.0%), and professional diplomas (11.7%), respectively. The sample-set covered managerial staff from diverse experience backgrounds of 5 years and less (13.6%), 6–10 years (52.7%), 11–15 years (19.2%), 15–20 years (11.7%), and 21 years and above (2.8%). Most of the respondents were functional and general managers of e-commerce companies, 47.3 and 43.8%, respectively; however, only 8.8% were CEO.

**TABLE 1:** Demographics of the respondents.

**Specification**	**Number**	**Percentage (%)**
**Gender**		
Male	160	50.5
Female	157	49.5
**Education**		
Bachelor and below	48	15.1
Masters	178	56.2
Doctorate	54	17.0
Professional Diploma	37	11.7
**Experience**		
5 years and less	43	13.6
6–10 years	167	52.7
11–15 years	61	19.2
15–20 years	37	11.7
21 years and above	9	2.8
**Job role**		
CEO	28	8.8
Functional Manager	150	47.3
General Manager	139	43.8

The descriptive statistics (mean and SD), reliability, and validity measures are illustrated in Table 2. Mean values for each construct fell between 3.920 and 3.748, and the SD fell between 1.020 and 1.091. This study used a five-point Likert scale; therefore, results fall within the range.

**TABLE 2:** Measurement model and descriptive statistics.

**Constructs**	**Code**	**FD**	**Cronbach α**	**CR**	**AVE**	**M**	**SD**
Good governance			0.91	0.933	0.735	3.843	1.020
	GG1	0.891					
	GG2	0.847					
	GG3	0.872					
	GG4	0.813					
	GG5	0.863					
Social reforms			0.912	0.93	0.657	3.748	1.050
	SR1	0.817					
	SR2	0.802					
	SR3	0.845					
	SR4	0.714					
	SR5	0.877					
	SR6	0.793					
	SR7	0.818					
Economic policies			0.891	0.92	0.699	3.92	1.084
	EP1	0.893					
	EP2	0.83					
	EP3	0.82					
	EP4	0.752					
	EP5	0.877					
Sustainable digital economy			0.899	0.93	0.768	3.8855	1.091
	SDE1	0.879					
	SDE2	0.879					
	SDE3	0.865					
	SDE4	0.882					

*FD, factor loadings; CR, construct reliability; AVE, average variance extracted; α, Cronbach alpha.*

**FIGURE 2:**
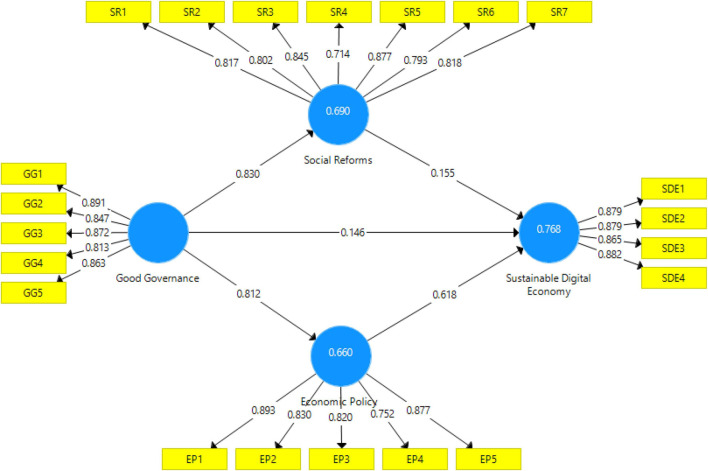
Measurement model outcomes.

The measurement model was used to assess the reliability and validity and the data set of the constructs through convergent and discriminant validity. The outcomes of the measurement model are given in Table 2 and Figure 2. The reliability of analysis access to what extent results are persistent and reliable over different scenarios. This study estimated the reliability of the constructs with Cronbach alpha and construct reliability (CR). Both Alpha and CR values fell above the minimum point of 0.70. Thus, the construct reliability is achieved. Factor loading above the minimum point 0.70 indicated the reliability of each measure in the construct; thus, no values below 0.70, and so measures reliability is also maintained. Talking about the convergent validity, all values of the average variance extracted should be not less than 0.50. As results indicated in Table 2, no value is below 0.50, confirming that convergent validity is attained.

The discernment validity is a source to measure the association or correlation between all variables. This study considered the criterion of Fornell and Larcker and the Heterotrait-Monotrait ratio. Fornell and Larcker ratio was accessed to find the discriminant validity where all diagonal values should be greater than the off-diagonal values. Values in bold illustrated in Tables 3, 4 show that the discriminant validity is achieved. Another measure is the heterotrait-monotrait (HTMT) ratio for discriminant validity. It measures the association between later variables with all other respective variables. The lower the value of HTMT describes, the higher the discriminant validity (Table 4).

**TABLE 3:** Fornell and Larcker criterion.

	**Economic**	**Good**	**Social**	**Sustainable**
	**policy**	**governance**	**reforms**	**digital economy**
Economic policy	**0.836**			
Good governance	0.812	**0.857**		
Social reforms	0.813	0.830	**0.811**	
Sustainable digital economy	0.823	0.777	0.779	**0.876**

**TABLE 4:** HTML ratio.

	**Economic**	**Good**	**Social**	**Sustainable**
	**policy**	**governance**	**reforms**	**digital economy**
Economic policy	–			
Good governance	0.065	–		
Social reforms	0.840	0.711	–	
Sustainable digital economy	0.693	0.827	0.824	–

The threshold for HTMT is 0.80 or 0.85, and values above 0.90 or near demonstrate the problem of discriminant validity. In the current research, all values are below 0.85. Therefore, the discriminant validity is maintained. All the measurements of discriminant validity confirmed the satisfactory discriminant validity in the data set.

**FIGURE 3:**
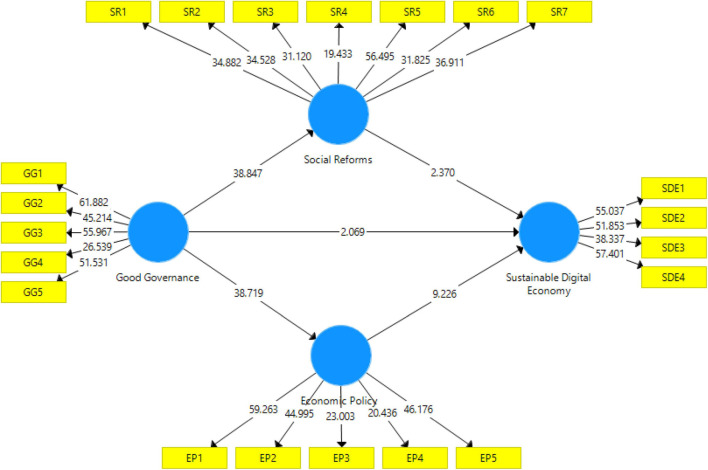
Structural model outcomes.

The structural model assessment was followed to test the hypothesis or path analysis between constructs (Figure 3). To test the hypothesis’s respective beta value (original sample), the *p*-value was considered. The analysis process took R^2^ to assess how much the independent variable is changed due to independent constructs, and the Q^2^ value accessed the predictive relevance of the model.

This study proposed seven hypotheses in total; among them five are direct and two are indirect. The first hypothesis confirmed a significant positive impact of good governance on a sustainable digital economy with β = 0.146; *p*−value = 0.019. Thus, *H1* meaningfully shows the sustainable digital economy. Good governance has positive impact on both the economic policies and social reforms under β = 0.812; *p*−value = 0.000 and β = 0.830; *p*−value = 0.000 respectively. Therefore, *H2* and *H3* were confirmed. Social reforms and economic policies have positive impact on sustainable digital economy under β = 0.155; *p*−value = 0.009 and β = 0.618; *p*−value = 0.000 respectively, thus *H4* and *H5* were accepted. In overview, all direct hypotheses were accepted. Results of the indirect hypothesis test confirmed that both mediating effects are accepted. Hypothesis six confirmed that social reforms partially mediate the relationship between good governance and sustainable digital economy with β = 0.502; *p*−value = 0.000. Likewise, economic policies partially mediate the relationship between good governance and a sustainable digital economy with β = 0.129; *p*−value = 0.009. Thus confirmed *H6* and *H7* (Table 5).

**TABLE 5:** Direct and indirect effects.

**H.**	**Paths**	**O**	**M**	**STDEV**	**T Stats**	***P* values**	**Results**	**Q2**	**R2**
H1	GG → SR	0.830	0.831	0.021	38.847	0.000	Supported	0.449	0.689
H2	GG → SDE	0.146	0.145	0.071	2.069	0.019	Supported	0.584	0.766
H3	GG → EP	0.812	0.813	0.021	38.719	0.000	Supported	0.457	0.659
H4	SR → SDE	0.155	0.156	0.065	2.37	0.009	Supported		
H5	EP → SDE	0.618	0.619	0.067	9.226	0.000	Supported		
H6	GG →SR → SDE	0.502	0.503	0.059	8.577	0.000	Supported		
H7	GG → EP → SDE	0.129	0.13	0.055	2.362	0.009	Supported		

*O, original sample or beta coefficient; M, sample mean; STDEV, standard deviation; H., hypothesis.*

**FIGURE 4:**
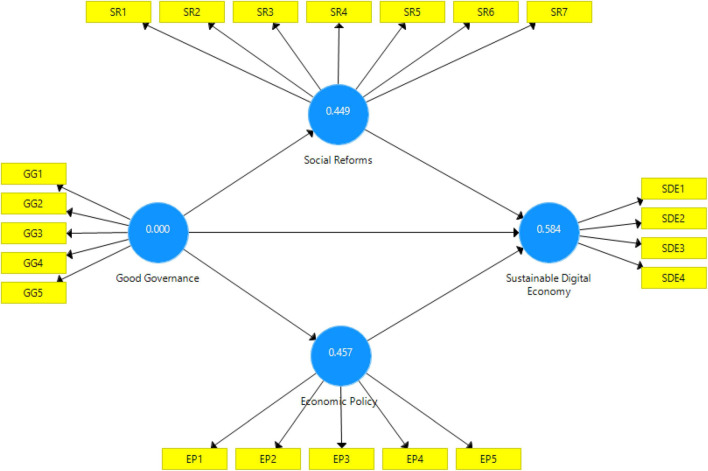
Predictive relevance of the model.

R^2^ values range between 0.689, 0.766, and 0.659; these coefficients indicated that (68.9, 76.6, and 65.9%) changes in independent variables (social reforms, sustainable digital economy, and economic policies) are due to independent constructs. Values for Q^2^, such as 0.02, 0.15, and 0.35, are classified as the model’s small, medium, and large predictive relevance. In short, the value for Q^2^ should be positive and non-zero. The values of Q^2^ (0.449, 0.584, and 0.457) confirmed a large predictive relevance of the model. Results for R^2^ are represented in Table 5 and for and Q^2^ in Table 5 and Figure 4.

## Discussion

Globalization and the digital economy have resulted in unprecedented growth in all corporate and public sectors and the emergence of a globally accessible market (Keping, 2018). Because a linked nation may change a digital economy with more effective and convenient private and public sectors, our study suggests that governments and private sectors must collaborate to develop a new digital ecosystem. The digital economy is altering traditional transactions and enabling new ones, both individually and collectively.

There is widespread agreement that governance mechanisms are required to correctly balance the potential benefits and hazards of digitalization while also ensuring the long-term viability of the digital economy (MacDonald and Hasmath, 2020). Therefore, this study offers several pathways for future improvements while focusing on economic policies and social norms. Various perspectives and ideas on the necessary governance strategies are required to establish digitalized economies, manage the processes, and mitigate the repercussions of digitalization were suggested by the current model of research.

Current research outcomes are homogenous and heterogenous to the previous body of knowledge and add in previous research by identifying predictors of a sustainable digital economy and analyzing the role of good governance in achieving a sustainable digital economy in China. The findings of current research showed that good governance always positively enhances the emergence of a sustainable digital economy. These findings are novel because no such study has investigated the current mechanism in this research strand. Additionally, this study examined the mediating role of China’s social reforms in comprehending this link and found that reforms enable states to make historic and significant achievements and rapid progress and continuous advances in the international stature of the economy. These research findings are well matched to the idea of Acheampong et al. (2018) and Chen et al. (2020). Additionally, this research investigated the mediating function of economic policies in the relationship between excellent governance and long-term economic policy. The outcomes of the current path are related to the previous body of knowledge. These studies have documented the role of economic policies in various contexts, but this is the first time it has been explored in this context. Social reforms and economic policies partially mediate the direct linkage between good governance and a sustainable digital economy. In overview, findings suggest that policymakers and decision-makers should use the outcomes of this study, particularly, the mediating function of social reforms and economic policy, to understand better the relationship between good governance and a sustainable digital economy. Therefore, these forces can be a potential source to mediate the association between good governance and sustainable digital economy, hence important for policymakers to improve the sustainable digital economy considering the economic policies, such as monetary, fiscal, taxation, effective tax collections, development of manufacturing, privatization, and macroeconomic stability. The right fit of economic policies and social reforms and good governance can help China make the economy digitally sustainable.

## Conclusion

There is widespread agreement that governance mechanisms are required to correctly balance the potential benefits and hazards of digitalization while also ensuring the long-term viability of the digital economy. Previous research is inclusive in concluding the predictors of a sustainable digital economy. Therefore, this study aims to look into the factors that influence the long-term viability of China’s digital economy. In addition, the significance of social reforms and economic policies in mediating the relationship between good governance and a sustainable digital economy was studied. The analysis technique used in this cross-sectional study was PLS-SEM. The information was gathered from 317 e-commerce business executives in China. The outcome research found that good governance positively impacts long-term sustainability, social reforms, and economic policies of the digital economy. In addition, digital economy of China has become more sustainable due to increased social changes and economic policies. Social reforms and economic policies somewhat mediated the relationship between excellent governance and a sustainable digital economy. These findings point to several measures to boost China’s digital economy’s long-term sustainability. These dynamics can mediate the relationship between good governance and a sustainable digital economy, which policymakers must enhance.

This study has some limitations. Firstly, this study considered the mediating roles of social reforms and economic policies; however, a moderating effect is short in the model that may produce more insightful outcomes if added. Secondly, current research focuses on the e-commerce industry. Thus, data were collected from e-commerce. Therefore, current findings cannot be generalized for other sectors and industries. Another contextual limitation is that the study is conducted in China. Therefore, country constraints are present in outcomes. Thirdly, the study is cross-sectional, and data were collected from a primary source (respondents). Individual responses are not as accurate as secondary data can be. Therefore, more research is called on using the secondary data in current research models. In addition, authors should segregate the individual mediating role of economic policies. A moderating role is missing in the current model. Thus, future research should add moderating role and investigate the current research model. Potential moderators can be economic conditions such as crisis periods or country inflation rates.

## Data Availability Statement

The original contributions presented in the study are included in the article/supplementary material, further inquiries can be directed to the corresponding author.

## Ethics Statement

All subjects gave their informed consent for inclusion before they participated in the study. The study was conducted in accordance with the Declaration of Helsinki, and the protocol was approved by the Xinjiang University, China.

## Author Contributions

TX conceived and designed the concept, wrote the manuscript. WQ collected the data and provided technical support. Both authors have read and agreed to the published version of the manuscript.

## Conflict of Interest

The authors declare that the research was conducted in the absence of any commercial or financial relationships that could be construed as a potential conflict of interest.

## Publisher’s Note

All claims expressed in this article are solely those of the authors and do not necessarily represent those of their affiliated organizations, or those of the publisher, the editors and the reviewers. Any product that may be evaluated in this article, or claim that may be made by its manufacturer, is not guaranteed or endorsed by the publisher.
